# Prognostic significance of receptor expression discordance between primary and recurrent breast cancers: a meta-analysis

**DOI:** 10.1007/s10549-021-06390-6

**Published:** 2021-10-06

**Authors:** Sho Shiino, Graham Ball, Binafsha M. Syed, Sasagu Kurozumi, Andrew R. Green, Hitoshi Tsuda, Shin Takayama, Akihiko Suto, Emad A. Rakha

**Affiliations:** 1 Division of Cancer and Stem Cells, School of Medicine, University of Nottingham, Nottingham, UK; 2 Department of Breast Surgery, National Cancer Center Hospital, Tokyo, Japan; 3 School of Science & Technology, John Van Geest Cancer Research Centre, Nottingham Trent University, Clifton Campus, Clifton Lane, Nottingham, UK; 4 Head of Clinical Research Division, Medical Research Centre, Liaquat University of Medical & Health Sciences, Jamshoro, Pakistan; 5 Department of Breast Surgery, International University of Health and Welfare, Narita, Japan; 6 Department of General Surgical Science, Gunma University Graduate School of Medicine, Maebashi, Japan; 7 Department of Basic Pathology, National Defense Medical College Hospital, Tokorozawa, Japan; 8 Department of Histopathology, Nottingham University Hospital NHS Trust, City Hospital Campus, Hucknall Road, Nottingham, NG5 1PB UK

**Keywords:** Breast cancer, Receptor discordance, Estrogen receptor, Progesterone receptor, Human epidermal growth factor receptor 2, Prognosis

## Abstract

**Purpose:**

This meta-analysis aimed to investigate whether receptor (estrogen receptor [ER], progesterone receptor [PR], and human epidermal growth factor receptor 2 [HER2]) discordances between primary and recurrent breast cancers affect patients’ survival.

**Methods:**

Search terms contained ER, PR, and HER2 status details in both primary and recurrent tumors (local recurrence or distant metastasis) in addition to survival outcome data (overall survival [OS] or post-recurrence survival [PRS]).

**Results:**

Loss of ER or PR in recurrent tumors was significantly associated with shorter OS as compared with receptor-positive concordance (hazard ratio [HR], 1.67; 95% confidence interval [% CI] 1.37–2.04; *p* < 0.00001 and HR, 1.45; 95% CI 1.21–1.75; *p* < 0.0001, respectively). Similar trends were observed in groups with only distant metastasis. Gain of ER was a significant predictor of longer PRS as compared with receptor-negative concordance (HR, 0.76; 95% CI 0.59–0.97; *p* = 0.03). Gain of PR was not a significant predictor of longer survival compared with receptor-negative concordance, but it could be related to better OS at distant metastasis. Both HER2 of loss and gain could be related to poor outcomes.

**Conclusion:**

This meta-analysis showed that receptor conversion in recurrent tumors may affect patient survival as compared with receptor concordance.

**Supplementary Information:**

The online version contains supplementary material available at 10.1007/s10549-021-06390-6.

## Introduction

Hormone receptors (estrogen receptor [ER] and progesterone receptor [PR]) and human epidermal growth factor receptor 2 [HER2] status in primary breast cancers (BC) not only provides prognostic information but it is also crucial for deciding on an effective treatment plan. Previous studies have reported discordance in these three biological markers between primary and recurrent tumors [1–4].

Some meta-analyses have clarified the rates of receptor discordance in these biological markers between primary and recurrent tumors [5, 6]. In a recent meta-analysis, ER, PR, and HER2 receptor discordance rates were reported as 19.3% (95% CI 15.8–23.4), 30.9% (95% CI 26.6–35.6), and 10.3% (95% CI 7.8–13.6), respectively [5]. Current guidelines recommend that patients with BC metastasis undergo biopsy or excisional biopsy to evaluate the status of these receptors [7, 8].

Previous studies have reported that receptor discordance may affect patient survival [9–11]. By contrast, there is no survival difference between patients with receptor discordance and those with receptor concordance [2]. Thus, no clear consensus on whether discordances of these receptors affect patient survival has been reached. Moreover, it remains unknown whether adjuvant therapy can affect the survival of patients with receptor discordance [5, 10, 11]. The aim of this study is to investigate whether discordance of receptors (ER, PR, and HER2) between primary and recurrent tumors affect the survival of patients with BC compared with receptor concordance using a meta-analytic approach.

## Methods

See Supplementary Text in the supplementary file for more information.

### Search strategy and eligibility criteria of articles

**Fig. 1:**
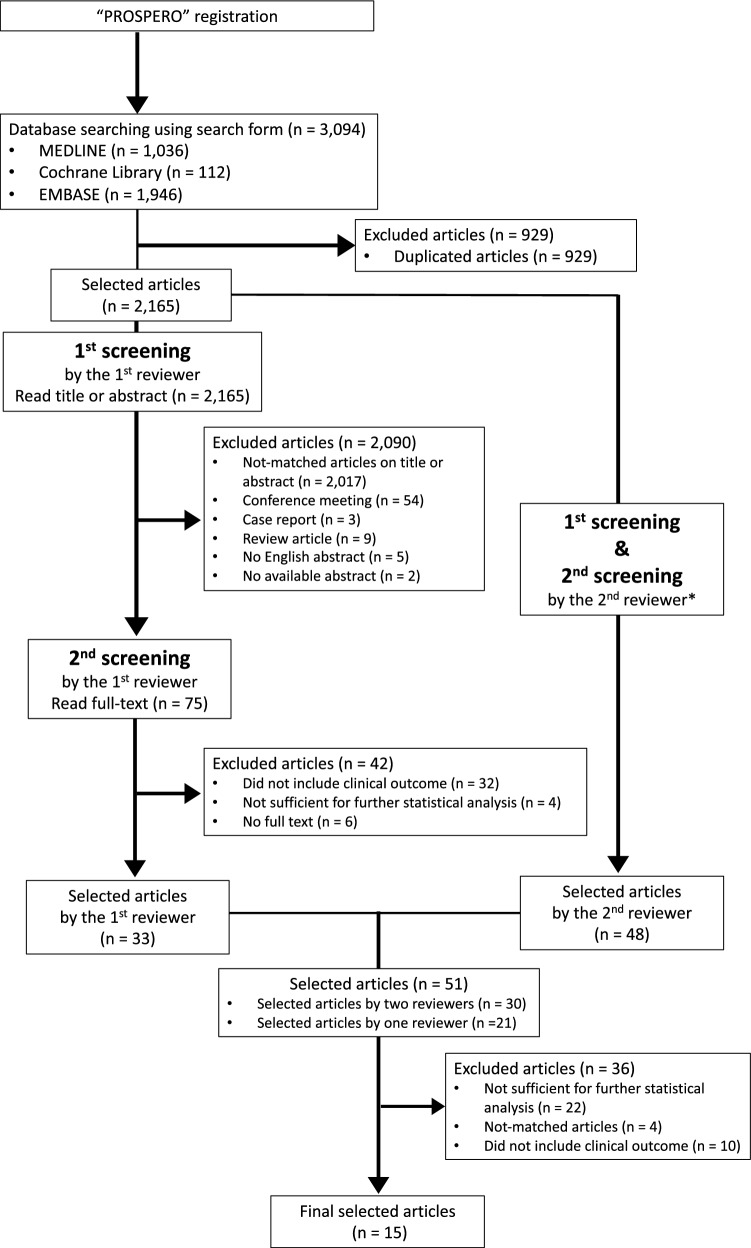
Flow chart of the selection procedure used for this meta-analysis. * The details are shown in Supplementary Fig. S1

We used preferred reporting items for systematic review and meta-analysis protocols (PRISMA-P, 2015) [12] to ensure a transparent and complete reporting of this research (Supplementary Table S1). The PICO elements (participants, interventions, comparators, and outcomes) for the clinical question, primary/secondary endpoints, inclusion/exclusion criteria, and subgroup analysis are shown in Supplementary Table S2. After searching the three electronic databases (MEDLINE, Cochrane library, and EMBASE), we excluded duplicated articles (Fig. 1).

A first screening was performed by reading the titles or abstracts. Studies meeting the following criteria were excluded in the first screening: (1) not relevant to our research objectives, (2) full title or abstract was unavailable, (3) gray literature (e.g., conference meeting abstracts, non-peer-reviewed literature), (4) review article, (5) case report, or (6) no abstract in English. A second screening was performed based on a full-text review of each article. In the second screening, we excluded articles that were unrelated to our topic, contained data unavailable for further statistical analysis, or the full text was unavailable.

All hits from the three databases were independently evaluated for selection or exclusion by two authors (SS and BMS) (Fig. 1 and Supplementary Fig. S1). Discrepancies of articles were discussed until consensus was reached and the finally selected articles were decided by the 1st reviewer (SS). Any discrepancies of selected articles between the two independent reviewers were evaluated using the kappa (*κ*) coefficient.

### Data extraction

We extracted some information from the finally selected articles as shown in Supplementary Table S2. We sent emails to each corresponding author of the final selected articles to obtain sufficient data. We allowed use of these missing data in the peer-reviewed literature for our meta-analysis, although these data were unpublished.

Tumors were pathologically classified based on the Tumor–Node–Metastasis (TNM) staging system [13]. ER and PR statuses for immunohistochemical (IHC) staining were considered positive if the cut-off was either ≥ 1% or ≥ 10% in reference to the European Society for Medical Oncology (ESMO) guidelines [14] or American Society of Clinical Oncology/College of American Pathologists (ASCO/CAP) guidelines [15, 16]. The HER2 expression level was mainly defined as positive according to the 2007 or 2013 ASCO/CAP guidelines [17, 18].

The definition of “receptor discordance” groups was defined as follows:

1. receptor loss (positive to negative, +/−), status positivity with primary breast tumor converted to status negativity with recurrent breast tumor or
2. receptor gain (negative to positive, −/+), status negativity with primary breast tumor converted to status positivity with recurrent breast tumor.

Cases in which no change in status occurred between primary and recurrent tumors were considered to have “receptor concordance,” which was either positive-to-positive (+/+) receptor concordance or negative-to-negative (−/−) receptor concordance. We compared the differences in survival between receptor-discordant groups and receptor-concordant groups as follows: receptor-loss (+/−) group vs. receptor-concordant (+/+) group or receptor-gain (−/+) group vs. receptor-concordant (−/−) group.

### Statistical analysis

Hazard ratio (HR) and 95% confidence interval (CI) were extracted from each study included in the final analysis. All results were calculated with a fixed-effects model. We also used a random-effects model to evaluate the difference in the heterogeneity between the two models. Statistical heterogeneity among studies was assessed using the *I*^2^ test. We evaluated *I*^2^ values according to the following definition: *I*^2^ > 50%, high heterogeneity; *I*^2^ 26–50%, moderate heterogeneity; and *I*^2^ 0–25%, low heterogeneity.

Forest plots were used to visualize heterogeneity among studies. Funnel plots were made to evaluate publication bias. A two-sided *p*-value of < 0.05 was considered statistically significant in both models. In cases where *I*^2^ values were much greater than 50% or significant, we performed sensitivity analysis or meta-regression analysis to evaluate reasons for the high heterogeneity. The risk of bias graph and bias summary were performed using Review Manager (RevMan) version 5.3. (The Cochrane Collaboration, Oxford, England) [19].

In cases where HR data or survival data were not explicitly stated in the literature, we extracted cumulative survival values using Engauge Digitizer software v12 from the relevant Kaplan–Meier survival curves. Moreover, we estimated the HR values from the extracted cumulative survival values using the Microsoft Excel spreadsheet reported previously [20]. We excluded groups with either fewer than ten total cases or fewer than five total events from our meta-analysis because we could not calculate data using our methods when the total number of cases of either the receptor-concordant or receptor-discordant group was too few.

## Results

### Characteristics of eligible studies

We identified 2,165 articles after excluding duplicated articles from the three electrical databases. After the first and second screenings, a total of 15 studies met all inclusion criteria. The discordance rate of selected articles between the two reviewers was 1.0% (21/2,165), and the *κ* value was 0.74, indicating a fair to good agreement. Some studies (4/15, 26.7%) included Stage IV BC at primary diagnosis [11, 21–23]. We excluded these four articles from our meta-analysis to minimize selection bias because survival data of patients with Stage IV BC are likely to skew meta-analysis results. Thus, 11 studies were finally selected (Table 1 and Supplementary Table S4) [24–34]. In these studies, diagnosis of a metastatic lesion was mainly performed by either core-needle biopsy or surgical excision. Some studies (4/11, 36.4%) included some cases that were diagnosed by fine-needle aspiration (FNA) of the metastatic lesion [25, 28, 30, 31]. We reasoned that these articles would not strongly affect survival data and therefore allowed their inclusion in our meta-analysis.

**Table 1:** The main characteristics of the studies used in our meta-analysis

Authors	Published year	Total cases with recurrence (n)	Total cases with details of statuses in both primary and recurrent tumors (n)			Country	Study design	Patient selection’s term (years)	Age at primary diagnosis: median (range)	Centralized laboratory testing	Review by pathologists	Receptor positive definition		
			ER	PR	HER2							ER, PR	HER2	FISH ratio
Curtit E, et al	2013	489	235	235	219	France	Retrospective	1998–2010	53 (26–89)	NA (single institution)	No	> 10%	3+ , 2+ (HER2 gene amplification by FISH)	HER2/CEP17 ratio > 2.0
Duchnowska R, et al	2012	120	120	119	119	Poland^c^	Retrospective	1996–2011	49^a^ (26–80)	Yes	Yes	AS 3–8 or ≥ 1% ^b^	3+ , 2+ (HER2 gene amplification by FISH)	HER2/CEP17 ratio ≥ 2.0
Lindström LS, et al	2012	1010	459	430	104	Sweden	Retrospective	1997–2007	NA	No (testing at some laboratories)	No	≥ 10%	3+ , 2+ (HER2 gene amplification by FISH)^f^	Two copies per cell
Hoefnagel LD, et al	2012	233	233	233	233	The Netherlands ^c^	Retrospective	1985–2009	53.9^a^ (25–93)	Yes	Yes	≥ 1% or ≥ 10%	3+ , 2+ (HER2 gene amplification by SISH)	SISH: HER2 copies/tumor cell nucleus ≥ 6
Niikura N, et al	2012	947	NA	NA	182	USA	Retrospective	1997–2008	48.2 (24–85)	Yes: 83 cases, No: 99 cases (46: review of slides stained by the other institution, 53 cases: slides were not available. Stained results were obtained by patients’ referral documents or communications)	Yes: 129 cases, No: 53 cases slides were not available. (Stained results were obtained by patients’ referral documents or communications)	≥ 10%	3+ and/or HER2 gene amplification by FISH	HER2/CEP17 ratio was ≥ 2.0
Meng X, et al	2016	627	627	627	503	China	Retrospective	2002–2016	44 (22–79)	Yes (single institution)	Yes	≥ 1%	3+ , 2+ (HER2 gene amplification by FISH)	HER2/CEP17 ratio > 2.2
Fujii K, et al	2017	70	70	69	70	Japan ^c^	Retrospective	1990–2012	54.5^a^ (NA)	Yes: all recurrent lesions and 59 primary lesions, No: 11 primary lesions	Yes	AS ≥ 3 and moderate-to-intense nuclear staining of ≥ 10%	3+ , 2+ (HER2 gene amplification by FISH)	HER2/CEP17 ratio > 2
Karlsson E, et al	2014	177	127	101	73	Sweden	Retrospective	2000–2011	NA	Yes	No	≥ 10%	3+ , 2+ (HER2 gene amplification by FISH)^f^	HER2/CEP17 ratio ≥ 2.0 or ≥ 4.0 HER2 gene copy number/tumor cell
Stueber TN, et al	2019	196	196	196	196	Germany	Retrospective	2000–2013	56.6^a^ (NA)	Yes (single institution)	No	(≥ 1%)	NA	NA
Shin HC, et al	2016	188	144	144	107	Korea	Retrospective	2000–2010	46 (24–71)	Yes (single institution)	No	≥ 10%	3+ , HER2 gene amplification by FISH	NA
Ju G, et al	2018	151	151	151	85	China^c^	Retrospective	2006–2016	56.6 (32–82)	NA (two institutions)	Yes	≥ 1%	3+ , 2+ (HER2 gene amplification by FISH)	HER2/CEP17 ratio ≥ 2.0 or HER2/CEP17 ratio < 2.0 and average HER2 copies/cells ratio ≥ 6.0

Authors	ER				PR				HER2			
	Concordance *n* (%)	Discordance *n* (%)	Loss *n* (%)	Gain *n* (%)	Concordance *n* (%)	Discordance *n* (%)	Loss *n* (%)	Gain *n* (%)	Concordance *n* (%)	Discordance *n* (%)	Loss *n* (%)	Gain *n* (%)
Curtit E, et al	195 (83.0)	40 (17.0)	29 (12.3)	11 (4.7)	166 (70.6)	69 (29.4)	52 (22.1)	17 (7.3)	211 (96.3)	8 (3.7)	6 (2.7)	2 (1.0)
Duchnowska R, et al	85 (70.8)	35 (29.2)	22 (18.3)	13 (10.9)	85 (71.4)	34 (28.6)	23 (19.3)	11 (9.3)	102 (85.7)	17 (14.3)	7 (5.9)	10 (8.4)
Lindström LS, et al	310 (67.6)	149 (32.4)	113 (24.6)	36 (7.8)	255 (59.3)	175 (40.7)	142 (33.0)	33 (7.7)	89 (85.6)	15 (14.4)	9 (8.6)	6 (5.8)
Hoefnagel LD, et al	198 (85.0)^d^ 209 (89.7) ^e^	35 (15.0) ^d^ 24 (10.3) ^e^	23 (9.9) ^d^ 17 (7.3) ^e^	12 (5.2) ^d^ 7 (3.0) ^e^	157 (67.4) ^d^ 163 (70.0) ^e^	76 (32.6) ^d^ 70 (30.0) ^e^	49 (21.0) ^d^ 58 (24.9) ^e^	27 (11.6) ^d^ 12 (5.1) ^e^	221 (94.8)	12 (5.2)	6 (2.6)	6 (2.6)
Niikura N, et al	NA	NA	NA	NA	NA	NA	NA	NA	139 (76.4)	43 (23.6)	43 (23.6)	NA
Meng X, et al	462 (73.7)	165 (26.3)	106 (16.9)	59 (9.4)	414 (66.0)	213 (34.0)	158 (25.2)	55 (8.8)	448 (89.1)	55 (10.9)	22 (4.4)	33 (6.5)
Fujii K, et al	55 (78.6)	15 (21.4)	9 (12.8)	6 (8.6)	44 (63.8)	25 (36.2)	15 (21.7)	10 (14.5)	63 (90.0)	7 (10.0)	0 (0)	7 (10.0)
Karlsson E, et al	109 (85.8)	18 (14.2)	15 (11.8)	3 (2.4)	61 (60.4)	40 (39.6)	30 (29.7)	10 (9.9)	66 (90.4)	7 (9.6)	4 (5.5)	3 (4.1)
Stueber TN, et al	175 (89.3)	21 (10.7)	19 (9.7)	2 (1.0)	153 (78.1)	43 (21.9)	34 (17.3)	9 (4.6)	181 (92.3)	15 (7.7)	5 (2.6)	10 (5.1)
Shin HC, et al	118 (82.0)	26 (18.0)	16 (11.1)	10 (6.9)	108 (75.0)	36 (25.0)	25 (17.4)	11 (7.6)	96 (89.7)	11 (10.3)	3 (2.8)	8 (7.5)
Ju G, et al	116 (76.8)	35 (23.2)	22 (14.6)	13 (8.6)	102 (67.5)	49 (32.5)	30 (19.9)	19 (12.6)	61 (71.8)	24 (28.2)	14 (16.5)	10 (11.7)

*AS* Allred score, *CEP* chromosome enumeration probe, *ER* estrogen receptor, *FISH* fluorescence in situ hybridization, *HER2* human epidermal growth factor receptor 2, *NA* not available, *PR* progesterone receptor, *SISH* silver in situ hybridization

^a^Mean age

^b^The results for AS and for ≥ 1% staining showed the same results

^c^Multi-institutional study

^d^1% threshold (The data were available on their previous study: Hoefnagel LD, et al. Receptor conversion in distant breast cancer metastases. *Breast Cancer Res.* 2010;12:R75.)

^e^10% threshold

^f^FISH was performed for both of HER2 2+ and HER2 3+

We evaluated these 11 remaining articles using the Risk of Bias Assessment tool for Non-randomized Studies (RoBANS) (Supplementary Text, Supplementary Fig. S2A and S2B). In each domain group, *κ*-values for the agreement between two reviewers were either excellent or fair to good as shown in Supplementary Fig. S2C. Of the 11 studies included, the number of articles that had performed overall survival (OS) analysis or post-recurrence survival (PRS) analysis was summarized in Supplementary Table S5.

Centralized laboratory testing was performed in 8 articles (8/11: 72.7%). The details were as follows: 6 articles, testing for all samples; 1 article, testing for 45.6% of total cases (83/182 cases); and 1 article, testing for 84.3% of total cases (59/70). The median discordant rates for the receptor statuses were 19.7% (range, 10.7–32.5%) for ER, 32.5% (21.9–40.7%) for PR, and 10.3% (3.7–28.2%) for HER2. The threshold definition of both ER and PR was either ≥ 1% (4/11, 36.4%) [24, 27, 29, 34] or ≥ 10% (7/11, 63.6%) [24–26, 28, 30, 31, 33]. Of these, one study investigated a survival difference effect on OS of simultaneously ≥ 10% and ≥ 1% [24]. Another study did not clearly mention the definition of hormone receptor positivity [32]. The positive definition for HER2/CEP17 ratio was mainly decided as either ≥ 2.2 or ≥ 2.0.

In the selected articles, survival data were available in six studies (6/11, 54.5%) each for (1) local recurrences and distant metastases [25, 28, 30–32, 34] or (2) only distant metastases [24–27, 29, 33]. One study investigated the survival difference of both (1) and (2) [25].

### Influence of receptor discordance on OS

**Fig. 2:**
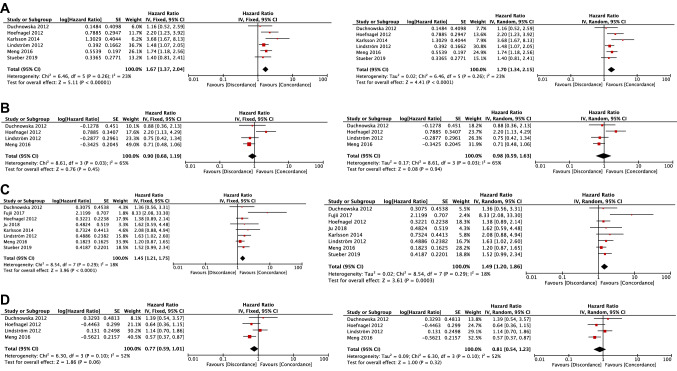
Forest plots comparing overall survival between receptor-loss/gain groups and receptor-concordant groups. **A** Comparison of OS between ER-loss (+/−) group and ER-concordant (+/+) group. **B** Comparison of OS between ER-gain (−/+) group and ER-concordant (−/−) group. **C** Comparison of OS between PR-loss (+/−) group and PR-concordant (+/+) group. **D** Comparison of OS between PR-gain (−/+) group and PR-concordant (−/−) group. *ER* estrogen receptor, *OS* overall survival, *PR* progesterone receptor

The ER-loss (+/−) and PR-loss (+/−) groups were significantly associated with shorter OS compared with the ER-concordant (+/+) and PR-concordant (+/+) groups, respectively ([Fixed-effects model] ER groups: HR, 1.67; 95% CI 1.37–2.04; *p* < 0.00001; PR groups: HR, 1.45; 95% CI 1.21–1.75; *p* < 0.0001, [Random-effects model] ER groups: HR, 1.70; 95% CI 1.34–2.15; *p* < 0.0001; PR groups: HR, 1.49; 95% CI 1.20–1.86; *p* = 0.0003) (Fig. 2A, C).

The ER-gain (−/+) and PR-gain (−/+) groups were not significantly associated with OS when compared with the ER-concordant (−/−) and PR-concordant (−/−) groups ([Fixed-effects model] ER groups: HR, 0.90; 95% CI 0.68–1.19; *p* = 0.45; PR groups: HR, 0.77; 95% CI 0.59–1.01; *p* = 0.06, [Random-effects model] ER groups: HR, 0.98; 95% CI 0.59–1.63; *p* = 0.94; PR groups: HR, 0.81; 95% CI 0.54–1.23; *p* = 0.32) (Fig. 2B and D).

Data regarding HER2-loss analysis were available in only 2/11 studies [27, 32]. However, these data were insufficient to perform a meta-analysis according to our methods. Likewise, data regarding HER2-gain analysis were also available in 2/11 studies [27, 32]. We were able to extract HR data from only one study according to our methods (HR, 2.17; 95% CI 0.82–5.74; *p* = 0.12) [27].

### Influence of receptor discordance on PRS

**Fig. 3:**
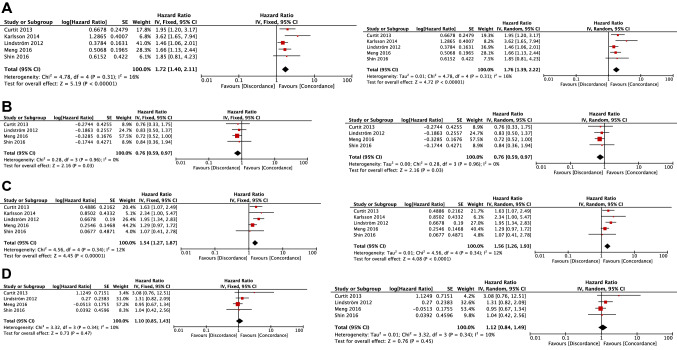
Forest plots comparing post-recurrence survival between receptor-loss/gain group and receptor-concordant group. **A** Comparison of PRS between ER-loss (+/−) group and ER-concordant (+/+) group. **B** Comparison of PRS between ER-gain (−/+) group and ER-concordant (−/−) group. **C** Comparison of PRS between PR-loss (+/−) group and PR-concordant (+/+) group. **D** Comparison of PRS between PR-gain (−/+) group and PR-concordant (−/−) group. *ER* estrogen receptor, *PR* progesterone receptor, *PRS* post-recurrence survival

The ER-loss (+/−) and PR-loss (+/−) groups were significantly associated with shorter PRS compared with the ER-concordant (+/+) and PR-concordant (+/+) groups, respectively ([Fixed-effects model] ER groups: HR, 1.72; 95% CI 1.40–2.11; *p* < 0.00001; PR groups: HR, 1.54; 95% CI 1.27–1.87; *p* < 0.00001, [Random-effects model] ER groups: HR, 1.76; 95% CI 1.39–2.22; *p* < 0.00001; PR groups: HR, 1.56; 95% CI 1.26–1.93; *p* < 0.0001) (Fig. 3A and C).

The ER-gain (−/+) group was significantly associated with longer PRS compared with the ER-concordant (−/−) group ([Fixed-effects model] HR, 0.76; 95% CI 0.59–0.97; *p* = 0.03, [Random-effects model] HR, 0.76; 95% CI 0.59–0.97; *p* = 0.03) (Fig. 3B). There was no significant difference in PRS between the PR-gain (−/+) and PR-concordant (−/−) groups ([Fixed-effects model] HR, 1.10; 95% CI 0.85–1.43; *p* = 0.47, [Random-effects model] HR, 1.12; 95% CI 0.84–1.49; *p* = 0.45) (Fig. 3D).

HER2-loss data were available in 3/11 studies [26, 28, 33]. Only one study was eligible for comparison between these two groups (HR, 2.78; 95% CI 1.61–4.76; *p* = 0.0002) [28]. HER2-gain data were available in two studies [26, 33], but the data were insufficient for meta-analysis.

### Influence on survival in patients with distant metastasis

**Fig. 4:**
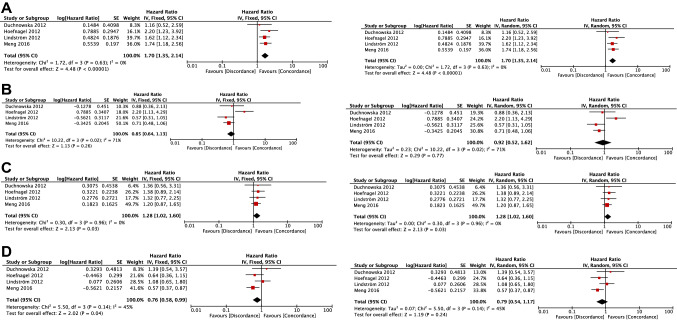
Forest plots comparing overall survival between receptor-loss/gain groups and receptor-concordant group in the subgroup of distant metastasis. **A** Comparison of OS between ER-loss (+/−) group and ER-concordant (+/+) group. **B** Comparison of OS between ER-gain (−/+) group and ER-concordant (−/−) group. **C** Comparison of OS between PR-loss (+/−) group and PR-concordant (+/+) group. **D** Comparison of OS between PR-gain (−/+) group and PR-concordant (−/−) group. *ER* estrogen receptor, *OS* overall survival, *PR* progesterone receptor

In the subgroup with only distant metastasis not including local recurrences, the OS of both ER-loss (+/−) and PR-loss (+/−) groups was significantly shorter than those of ER-concordant (+/+) and PR-concordant (+/+) groups, respectively ([Fixed-effects model] ER groups: HR, 1.70; 95% CI 1.35–2.14; *p* < 0.00001; PR groups: HR, 1.28; 95% CI 1.02–1.60; *p* = 0.03, [Random-effects model] ER groups: HR, 1.70; 95% CI 1.35–2.14; *p* < 0.00001; PR groups: HR, 1.28; 95% CI 1.02–1.60; *p* = 0.03) (Fig. 4A and C).

There was no significant difference in the OS between the ER-gain (−/+) and ER-concordant (−/−) groups ([Fixed-effects model] HR, 0.85; 95% CI 0.64–1.13; *p* = 0.26, [Random-effects model] HR, 0.92; 95% CI 0.52–1.62; *p* = 0.77) (Fig. 4B).

The PR-gain (−/+) group was significantly associated with longer OS compared with the PR-concordant (−/−) group (HR, 0.76; 95% CI 0.58–0.99; *p* = 0.04) in the fixed-effects model but was not significant in the random-effects model (HR, 0.79; 95% CI 0.54–1.17; *p* = 0.24) (Fig. 4D).

### Subgroup analysis according to the threshold (1% or 10%) of hormone receptor positivity

The ER-loss (+/−) and PR-loss (+/−) groups were significantly associated with shorter OS compared with the ER-concordant (+/+) and PR-concordant (+/+) group regardless of the definition of hormone receptor positivity and type of model (Supplementary Fig. S3A and S3C).

In subgroup analysis for the 1% and 10% thresholds, the ER-gain (−/+) group was not associated with OS compared with the ER-concordant (−/−) group in both of the models, respectively (Supplementary Fig. S3B). Meanwhile, in the subgroup analysis of 1% threshold, the OS of PR-gain (−/+) group was significantly longer than those of the PR-concordant (−/−) group in the fixed-effects model (HR, 0.66; 95% CI 0.47–0.90; *p* = 0.01) but not significant in the random-effects model (HR, 0.68; 95% CI 0.45–1.03; *p* = 0.07) (Supplementary Fig. S3D).

There was no significant difference between the two subgroups in the ER-loss analysis ([Fixed-effects model] *I*^2^ = 0%; *p* = 0.83, [Random-effects model] *I*^2^ = 0%; *p* = 0.81). There was no significant difference between the two subgroups in the ER-gain analysis ([Fixed-effects model] *I*^2^ = 0%; *p* = 0.49, [Random-effects model] *I*^2^ = 0%; *p* = 0.43). Contrarily, there was a significant difference between the two subgroups in the PR-loss analysis ([Fixed-effects model] *I*^2^ = 81.8%; *p* = 0.02, [Random-effects model] *I*^2^ = 77.2%; *p* = 0.04). There was a significant difference between the two subgroups in the PR-gain analysis in the fixed-effects model (*I*^2^ = 79.0%; *p* = 0.03) but not in the random-effects model (*I*^2^ = 69.5%; *p* = 0.07).

### Subgroup analysis according to the assessment of risk of bias

There was not a significant difference between the high- and low-risk-of-bias groups in the analysis of the OS in both models (Supplementary Fig. S4).

In the subgroup analysis of the low-risk-of-bias group, both the ER-loss (+/−) and PR-loss (+/−) groups had a significantly shorter OS than those of the ER-concordant (+/+) and PR-concordant (+/+) groups in the two models, respectively. Meanwhile, in the subgroup analysis of the low-risk-of-bias group, the PR-gain (−/+) group had a significantly longer OS than the PR-concordant (−/−) group in the fixed-effects model (HR, 0.74; 95% CI 0.56–0.97; *p* = 0.03), but there was not a significant difference in the random-effects model (HR, 0.74; 95% CI 0.48–1.15; *p* = 0.19).

### Subgroup analysis according to the study area

There was no significant difference between the Western group and Asian group in the receptor-loss analysis ([Fixed-effects model] ER-loss: *I*^2^ = 0%; *p* = 0.82, PR-loss: *I*^2^ = 0%; *p* = 0.51, [Random-effects model] ER-loss: *I*^2^ = 0%; *p* = 0.96, PR-loss: *I*^2^ = 0%; *p* = 0.53; Supplementary Fig. S5).

In the receptor-gain analysis, there was no significant difference between the two subgroups ([Fixed-effects model] ER-gain: *I*^2^ = 61.0%; *p* = 0.11, PR-gain: *I*^2^ = 70.5%; *p* = 0.07, [Random-effects model] ER-gain: *I*^2^ = 23.3%; *p* = 0.25, PR-gain: *I*^2^ = 63.9%; *p* = 0.10). Statistical heterogeneity was high in the PR-gain analysis in the two models.

### Evaluation of adjuvant therapy and changing treatment based on the status of each receptor

In the 11 selected articles, the median rate of adjuvant therapy was observed in 42.9% (range 25.8–84.1%), 57.9% (range 18.1–92.5%), and 8.1% (range 2.9–41.8%) for hormonal therapy, chemotherapy, and HER2-targeted therapy, respectively (Supplementary Table S4). Of the 11 studies analyzed herein, adjuvant therapy could affect receptor discordance as follows: hormonal therapy and/or chemotherapy, 5/11 studies (45.5%); HER2-targeted therapy, 1/11 studies (9.1%). In the selected articles, we could not evaluate the changing rate of treatment according to receptor conversion in recurrent tumors due to insufficient data.

### Evaluation of heterogeneity and publication bias

Statistical heterogeneity was mainly low to moderate in the total analysis of receptor-loss groups but was relatively moderate to high in the total analysis of receptor-gain groups. Particularly, heterogeneity in the ER-gain analysis on OS was significantly observed (Fig. 2B). One study showed poor survival in ER-gain groups with recurrent tumors [24], whereas the other three studies showed the tendency of better survival [25, 27, 29]. Thus, a meta-analysis was conducted in cases excluding the discrepant study, an analysis defined as “ER-gain’ (−/+) group vs. ER-concordant’ (−/−) group” (Supplementary Table S6). Statistical heterogeneity was not observed (*I*^2^ = 0%; *p* = 0.91), and the OS of the ER-gain’ (−/+) group was marginally associated with better OS than that of the ER-concordant’ (−/−) group (HR, 0.74; 95% CI 0.54–1.01; *p* = 0.06).

Meanwhile, in the PR-gain analysis on OS, statistical heterogeneity was marginally high (Fig. 2D). We performed meta-regression analysis to investigate whether the HR of PR-gain (−/+) could influence the rates of the other variables in the primary tumors for each article. The rate of HER2-positivity in the primary tumor was significantly correlated with the HR of PR-gain (−/+) on the OS analysis (*p* = 0.034) (Supplementary Table S7 and Supplementary Fig. S6).

We were unable to evaluate the risks of publication bias with Egger’s test because each analysis contained fewer than ten studies. Funnel plots on each analysis are summarized in Supplementary Fig. S7.

## Discussion

In the current meta-analysis study, we showed that loss of either ER or PR in recurrent tumors was significantly associated with poor prognosis in terms of OS and PRS. Gain of ER tends to be associated with better outcome in terms of longer PRS but not OS compared with receptor-negative primary and recurrent tumors. HER2 loss and gain could be related to poor outcome as compared with receptor-positive and receptor-negative concordance, respectively. As Li et al. reported a meta-analysis on the prognostic impact of receptor discordance after neoadjuvant chemotherapy, it did not investigate patient survival in cases of recurrent tumors [35]. To our knowledge, this is the first report to investigate whether receptor discordance affects the survival of patients with recurrent tumors using the meta-analytic method.

In the analysis of distant metastasis (Fig. 4), trends for each result were similar to the total analysis of OS (Fig. 2). Furthermore, statistical heterogeneity tended to be lower compared with those analyses. It is estimated that there are survival differences between local and distant in some previous reports [36, 37]. However, the reason for this could not be clarified herein because survival differences of patients that had only local recurrence not having distant metastases between receptor discordance and receptor concordance were unavailable.

In our meta-analysis, we found that significant heterogeneity between the 1% and 10% thresholds was found during PR analysis. The reason for statistical heterogeneity has not been clarified; however, previous studies have suggested that the rate of PR positivity is a more continuous variable as a prognostic factor [15, 38]. In particular, some studies have reported that cases with 1–10% PR expression behaved the same as those with receptor-negative disease [39–41]. Thus, the prognosis would be different according to the threshold definition.

Some studies have reported that adjuvant therapy could influence the receptor discordance in our selected articles. However, because our meta-analysis did not focus it as a primary endpoint, the relation between receptor discordance and adjuvant treatment requires further investigation. Meanwhile, some studies have reported a treatment strategy that was altered in reference to receptor changes in recurrent BC [1, 2, 42–44]. Moreover, discordant cases could lead to inappropriate hormone therapy treatments after the recurrence [2, 10] and treatment alteration based on receptor discordance could affect patient survival [29]. Those findings and our results highly recommended performing a biopsy of recurrent tumors to provide information for altering treatment strategies. However, performing a biopsy of all recurrent sites is invasive, costly, and cannot describe all recurrent sites. Liquid biopsy may be beneficial to detect tumor heterogeneity in circulating tumor cells [45, 46].

Discordance in those receptors has been reported due to analytical error, intratumoral heterogeneity [47], and cellular clonal evolution [48, 49]. The mechanisms of receptor discordance have been gradually elucidated [50]. Unfortunately, the molecular mechanisms for receptor discordance cannot be explained by the results of our study.

Some limitations of our study are as follows: (1) We could not perform a meta-analysis for HER2 discordance. The total number of HER2-discordant cases was too small in each study. A large number of studies will be required to verify these findings, especially for the HER2-gain group. Additionally, all studies were mainly based on 2007 or 2013 ASCO/CAP guidelines [17, 18] but not on the updated guidelines [51]. (2) In cases that we were not able to get HR and 95% CI data even if we inquired each corresponding author to get the data about HR and 95% CI on each article, we extracted those data using specialized software. This technique is commonly used to perform meta-analyses, but the calculated data are not the same as the original data, especially in cases of including more censored cases as most studies include censored data. Additionally, one article suggested no significant differences, but our calculations showed a significant association with poor survival [24]. (3) Each cohort contained different clinicopathological characteristics and different treatment options (adjuvant therapies or systemic therapies after recurrence), particularly between hormone receptor-positive and negative patients. Multivariate analytic results to adjust the confounding factors were mentioned in some original articles but not all articles. Thus, our conclusion regarding the effect of receptor discordance may not be robust as clinicopathological characteristics and treatment options are different among each cohort and our analysis could not fully adjust the confounding factors. In cases wherein clinicopathological characteristics are different among studies, a random-effects model results could be more appropriate than a fixed-effects model. Lim et al. have reported that the clinicopathological staging and survival outcomes are different between Asian and European groups [52]. Therefore, a random-effects model results would be more recommended in the subgroup analysis according to the study area. (4) Our meta-analysis included many studies performed over several decades, which could introduce bias because treatment strategies could vary with time.

In conclusion, in this meta-analysis, we have shown that receptor discordance between primary tumors and recurrent tumors could be associated with patient OS and PRS compared with receptor concordance. Analyzing receptor status to confirm the diagnosis of recurrent BC would provide information for altering treatment strategies based on either receptor-loss or receptor-gain status of recurrent tumors.

## Supplementary Information

Below is the link to the electronic supplementary material.

Supplementary file1 (DOCX 5446 KB)

## Abbreviations

ER: Estrogen receptor
PR: Progesterone receptor
HER2: Human epidermal growth factor receptor 2
BC: Breast cancer
PRISMA-P: Preferred reporting items for systematic review and meta-analysis protocols
PICO: Participants, interventions, comparators, and outcomes
PROSPERO: A prospective international register of systematic reviews
TNM: Tumor–Node–Metastasis
IHC: Immunohistochemical
ESMO: The European Society for Medical Oncology
ASCO/CAP: American Society of Clinical Oncology/College of American Pathologists
RoBANS: The Risk of Bias Assessment tool for Non-randomized Studies
HR: Hazard ratio
CI: Confidence interval
FNA: Fine-needle aspiration
OS: Overall survival
PRS: Post-recurrence survival
