# Supplementary material for: Prognostic significance of receptor expression discordance between primary and recurrent breast cancers: a meta-analysis
Source: Breast Cancer Res Treat. 2021 Oct 6;191(1):1–14. doi: 10.1007/s10549-021-06390-6 (PMC8758639; doi:10.1007/s10549-021-06390-6)
Supplement: Supplementary file 1 — Supplementary file1 (DOCX 5446 KB) [file 10549_2021_6390_MOESM1_ESM.docx]

**Supplementary Table S1.**

PRISMA-P 2015 checklist

*Details of either section, Table, Figure, or supplementary file are described in this column instead of line numbers.

| **Section/topic** | **#** | **Checklist item** | **Information reported** | | **Line number(s)*** |
| --- | --- | --- | --- | --- | --- |
|  |  |  | **Yes** | **No** |  |
| **ADMINISTRATIVE INFORMATION** | | | | | |
| **Title** | | | | | |
| Identification | 1a | Identify the report as a protocol of a systematic review |  |  | Described as the “title” |
| Update | 1b | If the protocol is for an update of a previous systematic review, identify as such |  |  |  |
| **Registration** | 2 | If registered, provide the name of the registry (e.g., PROSPERO) and registration number in the Abstract |  |  | Supplementary Text |
| **Authors** | | | | | |
| Contact | 3a | Provide name, institutional affiliation, and e-mail address of all protocol authors; provide physical mailing address of corresponding author |  |  | Described on the first page |
| Contributions | 3b | Describe contributions of protocol authors and identify the guarantor of the review |  |  | Described in the section of “Authors’ contributions” |
| **Amendments** | 4 | If the protocol represents an amendment of a previously completed or published protocol, identify as such and list changes; otherwise, state plan for documenting important protocol amendments |  |  | Supplementary Text |
| **Support** | | | | | |
| Sources | 5a | Indicate sources of financial or other support for the review |  |  | Described in the section of “Funding” |
| Sponsor | 5b | Provide name for the review funder and/or sponsor |  |  |  |
| Role of sponsor/funder | 5c | Describe roles of funder(s), sponsor(s), and/or institution(s), if any, in developing the protocol |  |  |  |
| **INTRODUCTION** | | | | | |
| **Rationale** | 6 | Describe the rationale for the review in the context of what is already known |  |  | Described in the “Introduction” session |
| **Objectives** | 7 | Provide an explicit statement of the question(s) the review will address with reference to participants, interventions, comparators, and outcomes (PICO) |  |  | Supplementary Table S2 |
| **METHODS** | | | | | |
| **Eligibility criteria** | 8 | Specify the study characteristics (e.g., PICO, study design, setting, time frame) and report characteristics (e.g., years considered, language, publication status) to be used as criteria for eligibility for the review |  |  | Supplementary Table S2 |
| **Information sources** | 9 | Describe all intended information sources (e.g., electronic databases, contact with study authors, trial registers, or other grey literature sources) with planned dates of coverage |  |  | Described in the “Methods” section, Supplementary Text, Table 1, Supplementary Table S2 |
| **Search strategy** | 10 | Present draft of search strategy to be used for at least one electronic database, including planned limits, such that it could be repeated |  |  | Supplementary Table S3 |

| **Section/topic** | **#** | **Checklist item** | **Information reported** | | **Line number(s)** |
| --- | --- | --- | --- | --- | --- |
|  |  |  | **Yes** | **No** |  |
| ***STUDY RECORDS*** | | | | | |
| Data management | 11a | Describe the mechanism(s) that will be used to manage records and data throughout the review |  |  | Supplementary Text |
| Selection process | 11b | State the process that will be used for selecting studies (e.g., two independent reviewers) through each phase of the review (i.e., screening, eligibility, and inclusion in meta-analysis) |  |  | Described in the “Methods” section, Figure 1, Supplementary Figure S1, Supplementary Table S2 |
| Data collection process | 11c | Describe planned method of extracting data from reports (e.g., piloting forms, done independently, in duplicate), any processes for obtaining and confirming data from investigators |  |  | Described in the “Methods” section, Supplementary Table S2 |
| **Data items** | 12 | List and define all variables for which data will be sought (e.g., PICO items, funding sources), any pre-planned data assumptions and simplifications |  |  | Described in the “Methods” section, Supplementary Table S2 |
| **Outcomes and prioritization** | 13 | List and define all outcomes for which data will be sought, including prioritization of main and additional outcomes, with rationale |  |  | Supplementary Table S2 |
| **Risk of bias in individual studies** | 14 | Describe anticipated methods for assessing risk of bias of individual studies, including whether this will be done at the outcome or study level, or both; state how this information will be used in data synthesis |  |  | Supplementary Text, Supplementary Figure S2 |
| ***DATA*** | | | | | |
| **Synthesis** | 15a | Describe criteria under which study data will be quantitatively synthesized |  |  | Described in the “Methods” section |
|  | 15b | If data are appropriate for quantitative synthesis, describe planned summary measures, methods of handling data, and methods of combining data from studies, including any planned exploration of consistency (e.g., *I* ^2^, Kendall’s tau) |  |  | Described in the “Methods” section |
|  | 15c | Describe any proposed additional analyses (e.g., sensitivity or subgroup analyses, meta-regression) |  |  | Described in the “Methods” section |
|  | 15d | If quantitative synthesis is not appropriate, describe the type of summary planned |  |  |  |
| **Meta-bias(es)** | 16 | Specify any planned assessment of meta-bias(es) (e.g., publication bias across studies, selective reporting within studies) |  |  | Described in the “Methods” section, Supplementary Figure S7 |
| **Confidence in cumulative evidence** | 17 | Describe how the strength of the body of evidence will be assessed (e.g., GRADE) |  |  | Supplementary Figure S2 |

**Supplementary Table S2.**

Protocol outline registered on PROSPERO

| **Type and method of review** | Meta-analysis | |
| --- | --- | --- |
| **Clinical question** | How does receptor (ER, PR, or HER2) discordance between primary and recurrent breast cancer impact on survival in patients with breast cancer compared to the receptor concordance? | |
| **PICO** | **P: Participants** | Patients with recurrent breast cancer that is available for ER, PR, and HER2 statuses both of primary and recurrent tumors |
|  | **I: Interventions** | Patients with breast cancer of receptor discordance between primary and recurrent breast tumors |
|  | **C: Comparators** | Patients with breast cancer of receptor concordance between primary and recurrent tumors |
|  | **O: Outcomes** | **Main outcome:** OS  **Additional outcome:** PRS, the discordant rate of each receptor status, changing treatment rate due to receptor discordance |
| **Endpoint** | **Primary** | OS: The term from the diagnostic date of primary tumors to the date of a patient’s death or last follow-up |
|  | **Secondary** | 1. PRS: The time from the date of diagnosis or biopsy of recurrent breast cancer to the date of death or last follow-up 2. The discordant rate of each receptor status 3. Changing treatment rate |
| **Searches** | The three electronic databases   1. MEDLINE 2. Cochrane Library 3. EMBASE | |
| **Inclusion criteria** | Prospective observational studies, observational cohort studies, and retrospective observational studies published in English including details of ER, PR, and HER2 statuses within primary or recurrent or metastatic tumor sites and also including survival outcome data  **Timing:** Not restricted | |
| **Exclusion criteria** | Duplicated articles | |
|  | **1^st^ screening**  **(Read title or abstract)** | 1. Not relevant to our research objectives 2. Full title or abstract was unavailable 3. Grey literature (conference meeting abstracts, non peer-reviewed literature) 4. Review article 5. Case report 6. No abstract in English |
|  | **2^nd^ screening**  **(Read full-text)** | 1. Unrelated to our topic 2. Unavailable for further statistical analysis 3. Unavailable full text |

| **Data extraction** | **General information** | Author names  Published year  Study design  Country  Total cases  Study term  Inclusion/exclusion criteria  Primary/secondary endpoint |
| --- | --- | --- |
|  | **Clinicopathological characteristics** | Age  Menopausal status  Tumor size  Lymph node status  Stage classification  Histologic type  Histologic grade^*1^  Receptor status of ER, PR, and HER2 in the primary/recurrent tumors  Scoring definition and technique for receptor statuses  Adjuvant treatment details  Metastatic sites  Treatment after recurrence  Obtained technique of recurrent tumor specimens |
|  | **Outcome data** | OS  PRS  Kaplan–Meier curves  Hazard ratio and 95%CI |
| **Subgroup analysis** | **Recurrent site** | 1. Locoregional recurrence 2. Distant metastasis |
|  | **Threshold of hormone receptor status^*2^** | 1. 1% 2. 10% |
|  | **Risk of bias^*2^** | 1. High-risk of bias group: The group include studies which have more than one ‘high risk’ of bias in the six domains. 2. Low-risk of bias group: The group include studies which have no ‘high risk’ of bias or have one ‘high risk’ of bias in the six domains. |
|  | **Study area^*2^** | 1. Western country 2. Asian country |

Abbreviations: BC, breast cancer; CI, confidential interval; ER, estrogen receptor; HER2, human epidermal growth factor receptor 2; OS, overall survival; PR, progesterone receptor; PRS, post-recurrence survival.

^*1^Histologic grade was mainly defined as the Nottingham grading system (Elston CW, Ellis IO. Pathological prognostic factors in breast cancer. I. The value of histological grade in breast cancer: experience from a large study with long-term follow-up. *Histopathology.* 1991;19:403-10.)

^*2^Those analyses were not mentioned in the original protocol.

**Supplementary Table S3.**

Search strategies for each electronic database

MEDLINE

|  | **Formula** |
| --- | --- |
| 1 | Exp Breast Neoplasms/ |
| 2 | ((breast or mammary) adj2 (cancer* or neoplasm* or carcinoma* or malignan* or "tumor*" or "tumour*")).mp. |
| 3 | ((breast or mammary) and "invasive ductal carcinoma").mp. |
| 4 | Exp Carcinoma, Ductal, Breast/ |
| 5 | 1 or 2 or 3 or 4 |
| 6 | Exp Recurrence/ |
| 7 | Exp Neoplasm Recurrence, Local/ |
| 8 | Exp Neoplasm Metastasis/ |
| 9 | (recurren* or recrudescence* or relaps* or metastas*).mp. |
| 10 | 6 or 7 or 8 or 9 |
| 11 | 5 and 10 |
| 12 | Exp Receptors, Estrogen/ |
| 13 | ("estrogen receptor*" or "oestrogen receptor*" or ER or ERalpha).mp. |
| 14 | 12 or 13 |
| 15 | Exp Receptors, Progesterone/ |
| 16 | ("progesterone receptor*" or "progestin receptor*" or PR or PgR).mp. |
| 17 | 15 or 16 |
| 18 | Exp Receptor, ErbB-2/ |
| 19 | ("human epidermal growth factor receptor 2" or HER2 or HER2neu or ERBB2).mp. |
| 20 | 18 or 19 |
| 21 | 14 or 17 or 20 |
| 22 | Exp Immunohistochemistry/ |
| 23 | Exp Staining and Labeling/ |
| 24 | (immunohistochemi* or IHC or stain* or label*).mp. |
| 25 | 22 or 23 or 24 |
| 26 | Exp In Situ Hybridization/ |
| 27 | ("in situ hybridization" or ISH or "fluorescence in situ hybridization" or FISH).mp. |
| 28 | 26 or 27 |
| 29 | (receptor adj5 status).mp. |
| 30 | 25 or 28 or 29 |
| 31 | ((conver* or chang* or shift* or alter* or gain or loss or increas* or decreas* or discord* or concord* or discrepan* or mismatch* or match* or inconsisten* or differ* or coincid* or compar*) adj5 (receptor* or ER or ERalpha or PR or PgR or HER2 or HER2neu or ERBB2)).mp. |
| 32 | Exp Survival/ |
| 33 | Exp Survival Analysis/ |
| 34 | Exp Survival Rate/ |
| 35 | Exp Mortality/ |
| 36 | Exp Prognosis/ |
| 37 | (survival or mortalit* or "death rate" or "kaplan meier" or prognos* or outcome*).mp. |
| 38 | 32 or 33 or 34 or 35 or 36 or 37 |
| 39 | 11 and 21 and 30 and 31 and 38 |
| 40 | 39 not neoadjuvant.mp. not meta-analysis.mp. |

Cochrane library

|  | **Formula** |
| --- | --- |
| 1 | MeSH descriptor: [Breast Neoplasms] |
| 2 | (breast OR mammary) NEAR/2 (cancer* OR neoplasm* OR carcinoma* OR malignan* OR "tumor*" OR "tumour*") |
| 3 | (breast OR mammary) AND "invasive ductal carcinoma" |
| 4 | MeSH descriptor: [Carcinoma, Ductal, Breast] |
| 5 | #1 OR #2 OR #3 OR #4 |
| 6 | MeSH descriptor: [Recurrence] |
| 7 | MeSH descriptor: [Neoplasm Recurrence, Local] |
| 8 | MeSH descriptor: [Neoplasm Metastasis] |
| 9 | recurren* OR recrudescence* OR relaps* OR metastas* |
| 10 | #6 OR #7 OR #8 OR #9 |
| 11 | #5 AND #10 |
| 12 | MeSH descriptor: [Receptors, Estrogen] |
| 13 | MeSH descriptor: [Estrogens] |
| 14 | MeSH descriptor: [Estrogen Receptor alpha] |
| 15 | "estrogen receptor*" OR "oestrogen receptor*" OR ER OR ERalpha |
| 16 | #12 OR #13 OR #14 OR #15 |
| 17 | MeSH descriptor: [Receptors, Progesterone] |
| 18 | MeSH descriptor: [Progesterone] |
| 19 | "progesterone receptor*" OR "progestin receptor*" OR PR OR PgR |
| 20 | #17 OR #18 OR #19 |
| 21 | MeSH descriptor: [Receptor, ErbB-2] |
| 22 | "human epidermal growth factor receptor 2" OR HER2 OR HER2neu OR ERBB2 |
| 23 | #21 OR #22 |
| 24 | #16 OR #20 OR #23 |
| 25 | MeSH descriptor: [Immunohistochemistry] |
| 26 | MeSH descriptor: [Staining and Labeling] |
| 27 | immunohistochemi* OR IHC OR stain* OR label* |
| 28 | #25 OR #26 OR #27 |
| 29 | MeSH descriptor: [in Situ Hybridization] |
| 30 | MeSH descriptor: [in Situ Hybridization, Fluorescence] |
| 31 | "in situ hybridization" OR ISH OR "fluorescence in situ hybridization" OR FISH |
| 32 | #29 OR #30 OR #31 |
| 33 | receptor NEAR/5 status |
| 34 | #28 OR #32 OR #33 |
| 35 | (conver* OR chang* OR shift* OR alter* OR gain OR loss OR increas* OR decreas* OR discord* OR concord* OR discrepan* OR mismatch* OR match* OR inconsisten* OR differ* OR coincid* OR compar*) NEAR/5 (receptor* OR ER OR ERalpha OR PR OR PgR OR HER2 OR HER2neu OR ERBB2) |
| 36 | MeSH descriptor: [Survival] |
| 37 | MeSH description: [Survival Analysis] |
| 38 | MeSH description: [Survival Rate] |
| 39 | MeSH description: [Mortality] |
| 40 | MeSH description: [Kaplan-Meier Estimate] |
| 41 | MeSH description: [Prognosis] |
| 42 | survival OR mortalit* OR "death rate" OR "kaplan meier" OR prognos* OR outcome* |
| 43 | #36 OR #37 OR #38 OR #39 OR #40 OR #41 OR #42 |
| 44 | #11 AND #24 AND #34 AND #35 AND #43 |
| 45 | #44 NOT neoadjuvant NOT meta-analysis |

EMBASE

|  | **Formula** |
| --- | --- |
| 1 | Exp breast tumor/ |
| 2 | ((breast or mammary) adj2 (cancer* or neoplasm* or carcinoma* or malignan* or "tumor*" or "tumour*")).mp. |
| 3 | ((breast or mammary) and "invasive ductal carcinoma").mp. |
| 4 | Exp breast carcinoma/ |
| 5 | Exp breast cancer/ |
| 6 | 1 or 2 or 3 or 4 or 5 |
| 7 | Exp tumor recurrence/ |
| 8 | Exp recurrent disease/ |
| 9 | Exp metastasis/ |
| 10 | Exp distant metastasis/ |
| 11 | Exp local metastasis/ |
| 12 | Exp regional metastasis/ |
| 13 | Exp bone marrow metastasis/ |
| 14 | Exp bone metastasis/ |
| 15 | Exp spine metastasis/ |
| 16 | Exp brain metastasis/ |
| 17 | Exp central nervous system metastasis/ |
| 18 | Exp liver metastasis/ |
| 19 | Exp lung metastasis/ |
| 20 | Exp pleura metastasis/ |
| 21 | Exp skin metastasis/ |
| 22 | Exp soft tissue metastasis/ |
| 23 | (recurren* or recrudescence* or relaps* or metastas*).mp. |
| 24 | 7 or 8 or 9 or 10 or 11 or 12 or 13 or 14 or 15 or 16 or 17 or 18 or 19 or 20 or 21 or 22 or 23 |
| 25 | 6 and 24 |
| 26 | Exp estrogen receptor/ |
| 27 | ("estrogen receptor*" or "oestrogen receptor*" or ER or ERalpha).mp. |
| 28 | 26 or 27 |
| 29 | Exp progesterone receptor/ |
| 30 | ("progesterone receptor*" or "progestin receptor*" or PR or PgR).mp. |
| 31 | 29 or 30 |
| 32 | Exp epidermal growth factor receptor 2/ |
| 33 | ("human epidermal growth factor receptor 2" or HER2 or HER2neu or ERBB2).mp. |
| 34 | 32 or 33 |
| 35 | 28 or 31 or 34 |
| 36 | Exp immunohistochemistry/ |
| 37 | Exp staining/ |
| 38 | (immunohistochemi* or IHC or stain* or label*).mp. |
| 39 | 36 or 37 or 38 |
| 40 | Exp in situ hybridization/ |
| 41 | ("in situ hybridization" or ISH or "fluorescence in situ hybridization" or FISH).mp. |
| 42 | 40 or 41 |
| 43 | (receptor adj5 status).mp. |
| 44 | 39 or 42 or 43 |
| 45 | ((conver* or chang* or shift* or alter* or gain or loss or increas* or decreas* or discord* or concord* or discrepan* or mismatch* or match* or inconsisten* or differ* or coincid* or compar*) adj5 (receptor* or ER or ERalpha or PR or PgR or HER2 or HER2neu or ERBB2)).mp. |
| 46 | Exp survival/ |
| 47 | Exp median survival time/ |
| 48 | Exp overall survival/ |
| 49 | Exp survival analysis/ |
| 50 | Exp survival rate/ |
| 51 | Exp survival time/ |
| 52 | Exp mortality/ |
| 53 | Exp cancer mortality/ |
| 54 | Exp mortality rate/ |
| 55 | Exp Kaplan Meier method/ |
| 56 | Exp prognosis/ |
| 57 | Exp cancer prognosis/ |
| 58 | (survival or mortalit* or "death rate" or "kaplan meier" or prognos* or outcome*).mp. |
| 59 | 46 or 47 or 48 or 49 or 50 or 51 or 52 or 53 or 54 or 55 or 56 or 57 or 58 |
| 60 | 25 and 35 and 44 and 45 and 59 |
| 61 | 60 not neoadjuvant.mp. not meta-analysis.mp. |

**Supplementary Table S4.**

Additional characteristics of the studies used in this meta-analysis

| **Authors** | **ER antibody (IHC)** | **PR antibody (IHC)** | **HER2 antibody (IHC)** | **FISH analysis for HER2 amplification** | **The purposes of study** | **Endpoint** | |
| --- | --- | --- | --- | --- | --- | --- | --- |
|  |  |  |  |  |  | **primary** | **secondary** |
| **Curtit E, et al.** | Two different ER antibodies: clone 6F11 (Novocastra), clone SP1 (Ventana Medical Systems) | Three different PR antibodies: clone PGR636 (DAKO), clone 16 (Novocastra), clone 1E2 (Ventana Medical Systems) | Two different HER2 antibodies: clone 4B5 (Ventana Medical Systems), clone CB11 (DAKO) | NA | The same as primary and secondary endpoint | Discordance in ER, PR, and HER2 status between primary BC and metastatic BC | Patients’ characteristics, OS, feasibility of histopathological evaluation in the metastatic setting, and predictive factors associated with receptor status discordance |
| **Duchnowska R, et al.** | Clone SP1 (DAKO) | Clone 636 (DAKO) | Clone 4B5 (Ventana Medical Systems) | NA | Compare of receptor statuses between primary and brain metastasis, assess the impact of factors influencing receptor conversion, and evaluate association of particular phenotypic changes with survival | NA | NA |
| **Lindström LS, et al.** | Monoclonal antibody–based biochemical methods (Abbott Laboratories) | | Three different HER2 antibodies (from 2000 to 2005): CB11 (Ventana Medical Systems), A485 (DAKO), and Ab-17 (Neomarkers),  Two antibodies (after March 2005): CB11(Ventana and Novocastra Leica), A485 (DAKO) | NA | Investigate whether receptor statuses change throughout tumor progression | NA | NA |
| **Hoefnagel LD, et al.** | M7047 (DAKO) | M3569 (DAKO) | RM-9103-S (Neomarkers) | INFORM (Ventana Medical Systems) | Evaluate the prognostic value of receptor conversion for ER and PR in distant non-bone BC metastases | NA | NA |
| **Niikura N, et al.** | NA | NA | NA (DAKO) | NA | Investigate the role of HER2 discordance and evaluate clinical factors that may influence such discordance | NA | NA |
| **Meng X, et al.** | NA | NA | NA | PathVysion HER-2 DNA probe kit (Abbott) | Compare the ER and PR between primary and recurrent BC, to investigate whether HR conversion influences the survival of BC patients, and to investigate the effect of HR conversion on salvage HT | NA | NA |
| **Fujii K, et al.** | Anti-ER (Roche) | Anti-PR (Roche) | Anti-HER2 (Agilent Technologies) | PathyVysion HER2 DNA probe kit (Abbott) | Compare ER, PR and HER2, and Ki67 between primary and recurrent BC, investigate correlations between changes in receptor status and patients’ survival | OS | NA |
| **Karlsson E, et al.** | 6F11 (Novocastra) and SP1 (DAKO) | Polyclonal PGR (Novocastra) and PGR636 (DAKO) | Antibody c-erb2 (DAKO) and Hercept-kit Sk001 | FISH Vysis-kit (Abbott) | Investigate characteristics of tumor related events, perform a comparative analysis of ER, PR, and HER2 and Ki67 between primary and recurrent BC | NA | NA |
| **Stueber TN, et al.** | NA | NA | NA | NA | Characterize receptor discordances between primary and recurrent BC, to evaluate influencing factors | NA | NA |
| **Shin HC, et al.** | NA | NA | NA | NA | Evaluate the receptor discordance between primary and recurrent BC, to examine the prognostic impact of discordant receptor status and phenotype after developing distant metastasis | NA | NA |
| **Ju G, et al.** | SP1 (Roche) | 1E2 (Roche) | 1E2 (Roche) | The Linked-Biotech Pathology HER2 DNA Probe kit (LBP) | Analyze the receptor discordance between primary and distant metastasis, investigate their impact on survival and treatment decisions | Discordance rates (ER, PR, HER2, and Ki67) between the primary and metastatic BC, the results of transformed treatment strategy | DFI, PFS, and OS |

|  | **Menopausal status^*1^: n (%)** | | | **Histologic type^*1^: n (%)** | | | | | |
| --- | --- | --- | --- | --- | --- | --- | --- | --- | --- |
| **Authors** | **Pre** | **post** | **Unknown** | **Intraductal** | **IDC** | **ILC** | **IDC + ILC** | **Others** | **Unknown** |
| **Curtit E, et al.** | NA | | | NA | | | | | |
| **Duchnowska R, et al.** | NA | | | 0  (0) | 98 (81.6) | 10  (8.3) | 2  (1.7) | 5  (4.2) | 5 (4.2) |
| **Lindström LS, et al.** | NA | | | NA | | | | | |
| **Hoefnagel LD, et al.** | NA | | | 0  (0) | 192 (82.4) | 20  (8.6) | 0 (0) | 20  (8.6) | 1  (0.4) |
| **Niikura N, et al.** | NA | | | NA | | | | | |
| **Meng X, et al.** | 443 (70.7) | 144 (23.0) | 40  (6.3) | NA | | | | | |
| **Fujii K, et al.** | 32 (45.7) | 38 (54.3) | 0 (0) | 1  (1.4) | 61 (87.1) | 2  (2.9) | 0  (0) | 6  (8.6) | 0 (0) |
| **Karlsson E, et al.** | NA | | | NA | | | | | |
| **Stueber TN, et al.** | 72  (36.7) | 124 (63.3) | 0 (0) | NA | | | | | |
| **Shin HC, et al.** | NA | | | NA | | | | | |
| **Ju G, et al.** | NA | | | 3  (2.0) | 137 (90.7) | 4  (2.7) | NA | 7  (4.6) | 0 (0) |

|  | **T factor^*1^: n (%)** | | | | | | **N factor^*1^: n (%)** | | | | | **Stage^*1^: n (%)** | | | | |
| --- | --- | --- | --- | --- | --- | --- | --- | --- | --- | --- | --- | --- | --- | --- | --- | --- |
| **Authors** | **Tis** | **T1** | **T2** | **T3** | **T4** | **Unknown** | **N0** | **N1** | **N2** | **N3** | **Unknown** | **0** | **I** | **II** | **III** | **Unknown** |
| **Curtit E, et al.** | NA | | | | | | NA | | | | | NA | | | | |
| **Duchnowska R, et al.** | NA | | | | | | NA | | | | | NA | | | | |
| **Lindström LS, et al.** | 67 (6.6)  ^*2^ | 407 (40.3)  ^*2^ | 396 (39.2)  ^*2^ | 95 (9.4)  ^*2^ | 29 (2.9)  ^*2^ | 16  (1.6)  ^*2^ | 785 (77.7)  ^*2^ | 199 (19.7)  ^*2^ | 6 (0.6)  ^*2^ | 5  (0.5)  ^*2^ | 15  (1.5)  ^*2^ | NA | 421 (41.7)  ^*2^ | 481 (47.6)  ^*2^ | 82 (8.1)  ^*2^ | 26  (2.6)  ^*2^ |
| **Hoefnagel LD, et al.** | 0 (0) | 73 (31.3) | 80 (34.3) | 12  (5.2) | | 68  (29.2) | 81 (34.8) | 119 (51.1) | | | 33  (14.1) | NA | | | | |
| **Niikura N, et al.** | NA | | | | | | NA | | | | | NA | | | | |
| **Meng X, et al.** | NA | | | | | | NA | | | | | 0 (0) | 110 (17.5) | 433 (69.1) | 84 (13.4) | 0  (0) |
| **Fujii K, et al.** | 1 (1.4) | 21  (30.0) | 33 (47.1) | 7  (10.0) | 5 (7.2) | 3  (4.3) | 29  (41.4) | 34 (48.5) | 3 (4.3) | 2  (2.9) | 2  (2.9) | 1 (1.4) | 14 (20.0) | 39 (55.7) | 13 (18.6) | 3 (4.3) |
| **Karlsson E, et al.** | NA | | | | | | NA | | | | | NA | | | | |
| **Stueber TN, et al.** | 0  (0) | 93  (47.4) | 84 (42.9) | 11  (5.6) | 8 (4.1) | 0  (0) | 92  (46.9) | 66 (33.7) | 25 (12.8) | 10  (5.1) | 3  (1.5) | NA | | | | |
| **Shin HC, et al.** | 0 (0) | 42  (29.2) | 92 (63.9) | 10  (6.9) | 0 (0) | 0  (0) | 58  (40.3) | 48 (33.3) | 14  (9.7) | 24 (16.7) | 0 (0) | 0 (0) | 23 (16.0) | 83 (57.6) | 38 (26.4) | 0 (0) |
| **Ju G, et al.** | 18  (11.9) | | 72 (47.7) | 5  (3.3) | | 56  (37.1) | 33 (21.9) | 36 (23.8) | 52  (34.4) | | 30  (19.9) | NA | | | | |

|  | **Histologic Grade^*1^: n (%)** | | | | **ER^*1^: n (%)** | | **PR^*1^: n (%)** | | **HER2^*1^: n (%)** | |
| --- | --- | --- | --- | --- | --- | --- | --- | --- | --- | --- |
| **Authors** | **1** | **2** | **3** | **Unknown** | **Positive** | **Negative** | **Positive** | **Negative** | **Positive** | **Negative** |
| **Curtit E, et al.** | NA | | | | 193 (82.1) | 42  (17.9) | 150 (63.8) | 85  (36.2) | 40  (18.3) | 179 (81.7) |
| **Duchnowska R, et al.** | 6^*3^ (5.0) | 41^*3^ (34.2) | 56^*3^ (46.6) | 17^*3^ (14.2) | 51 (42.5) | 69 (57.5) | 41 (34.5) | 78 (65.5) | 58 (48.7) | 61 (51.3) |
| **Lindström LS, et al.** | NA | | | | 329 (71.7) | 130 (28.3) | 251 (58.4) | 179 (41.6) | 29 (27.9) | 75 (72.1) |
| **Hoefnagel LD, et al.** | 8 (3.4) | 61 (26.2) | 161 (69.1) | 3 (1.3) | 174 (74.7)^*5^ 147 (63.1)^*6^ | 59 (25.3)^*5^ 86 (36.9)^*6^ | 173 (74.2) ^*5^ 129 (55.4) ^*6^ | 60 (25.8) ^*5^ 104 (44.6) ^*6^ | 47 (20.2) | 186 (79.8) |
| **Niikura N, et al.** | 2 (1.1)  ^*4^ | 35 (19.2)  ^*4^ | 137 (75.3)  ^*4^ | 8 (4.4)  ^*4^ | 93  (51.1) | 87  (47.8) | 65  (35.7) | 113  (62.1) | 182  (100) | 0  (0) |
| **Meng X, et al.** | NA | | | | 346  (55.2) | 281  (44.8) | 315  (50.2) | 312  (49.8) | 97  (19.3) | 406  (80.7) |
| **Fujii K, et al.** | 8  (11.4) | 35  (50.0) | 18  (25.7) | 9  (12.9) | 48  (68.6) | 22  (31.4) | 38  (55.1) | 31  (44.9) | 12  (17.1) | 58  (82.9) |
| **Karlsson E, et al.** | NA | | | | 102  (80.3) | 25  (19.7) | 60  (59.4) | 41  (40.6) | 18  (24.7) | 55  (75.3) |
| **Stueber TN, et al.** | 18  (9.2)^*3^ | 98  (50.0) ^*3^ | 59  (30.1) ^*3^ | 21  (10.7) ^*3^ | NA | | NA | | NA | |
| **Shin HC, et al.** | NA | | | | 74  (51.4) | 70  (48.6) | 50  (34.7) | 94  (65.3) | 29  (27.1) | 78  (72.9) |
| **Ju G, et al.** | 1  (0.7) | 32  (21.2) | 29  (19.2) | 89  (58.9) | 80  (53.0) | 71  (47.0) | 58  (38.4) | 93  (61.6) | 66  (57.4) | NA |

| **Authors** | **Metastatic sites** | **Adjuvant HT: n (%) ^*7^** | **Adjuvant CT: n (%) ^*7^** | **Adjuvant HER2-targeted therapy: n (%) ^*8^** | **Treatment after recurrence** | **Techniques for diagnosis in the recurrent tumors** |
| --- | --- | --- | --- | --- | --- | --- |
| **Curtit E, et al.** | Lung, bone, liver, CNS, soft tissues, others | 125  (53.2) | 136 (57.9) | 17^*11^  (8.1) | NA | Biopsy or excision |
| **Duchnowska R, et al.** | CNS only | 31^*9^ (25.8) | 47^*9^ (39.2) | 30 (25.2) | CT: 57 of 120 patients (47.5%) HT: 21 of 120 patients (17.5%) HER2-targeted therapy for patients with HER2-positive at metastases: Trastuzumab, 9 of 61 patients (14.8%); Lapatinib, 7 of HER2-positive 58 patients (11.5%); sequentially both, 3 patients (4.9%) | Excision |
| **Lindström LS, et al.** | Local recurrence, LN, lung, pleura, bone, liver, CNS, abdomen, skin, others | HT+CT: 99 (21.6)^*10^  HT only: 162 (35.3^)*10^  CT only: 111 (24.2)^*10^ | | NA | Approximately half the patients with ER-gain in recurrent tumors received HT. 24 of 104 patients (23.1%) received trastuzumab after recurrence. | Biopsy or FNA |
| **Hoefnagel LD, et al.** | Lung, liver, CNS, skin, GI | ER discordant group: NA (8.6), ER concordant group: NA (31.3)^*5^ | ER discordant group: NA (60.0), ER concordant group: NA (40.4) ^*5^ | NA | NA | Histologic specimens^*12^ |
| **Niikura N, et al.** | CW, regional LN, lung, pleura, bone, liver, CNS, skin, others | NA | 142 (78.0) | 76 (41.8) | 7 of 43 patients with HER2-negative metastases received HER2-targeted therapy (trastuzumab: 5 cases, lapatinib: 2 cases) after recurrence. | Biopsy or excision or FNA |
| **Meng X, et al.** | Soft tissues, lung, pleura, bone, liver, CNS, ovary, thyroid gland,  others | 313  (49.9) | 580  (92.5) | NA | Of the 121 patients whose HR changed positive to negative, 76 patients received HT and CT, and 45 patients received CT only. Of the 184 patients with HR-negative in both primary and recurrent BC, 32 patients received CT and HT and 152 patients received CT only. | Biopsy |
| **Fujii K, et al.** | Local recurrence, LN, bone, lung, pleura, liver, CNS, GI, others | HT only: 22  (31.4) | 57  (81.4) | 2  (2.9) | NA | Biopsy or excision or FNA |
| **Karlsson E, et al.** | Local recurrence, lung, pleura, bone, liver, CNS, skin, LN, GI, others | HT only: 48 (37.8)  HT+CT: 30 (23.6) | CT only: 23 (18.1)  HT+CT: 30 (23.6) | NA | NA | Biopsy or excision or FNA |
| **Stueber TN, et al.** | Local recurrence, lung, pleura, bone, bone marrow, liver, CNS, skin, LN, contralateral breast, peritoneum, others | 165  (84.1) | 106  (54.1) | NA | NA | Biopsy |
| **Shin HC, et al.** | Lung, liver, bone, contralateral LN, multiple sites | 69  (47.9)^*9^ | 129  (89.6)^*9^ | 5  (4.7)^*9^ | NA | Biopsy or excision |
| **Ju G, et al.** | Local recurrence, lung, pleura, bone, liver, skin, ovary,  ascites, thoracic wall, LN | NA | NA | NA | NA | Biopsy |

Abbreviations: BC, breast cancer; CT, chemotherapy; CNS, central nervous system; CW, chest wall; DFI, disease-free interval: the time from primary surgery to the confirmation of recurrence; ER, estrogen receptor; FISH, fluorescence *in situ* hybridization; FNA, fine needle aspiration; GI, gastrointestinal; HER2, human epidermal growth factor receptor 2; HR, hormone receptor; HT, hormonal therapy; IBTR, ipsilateral breast tumor recurrence; IDC, invasive ductal carcinoma; IHC, immunohistochemical staining; ILC, invasive lobular carcinoma; LN, lymph node; NA, not available; OS, overall survival; PFS, progression-free survival: the time from the diagnosis of recurrence to the progression of 1^st^ line treatment; PR, progesterone receptor.

^*1^Diagnosis of primary tumor

^*2^Number and proportion in total cases with recurrence

^*3^Details of either histologic grade or nuclear grade were not available.

^*4^Nuclear grade

^*5^1% threshold

^*6^10% threshold

^*7^Proportion in total cases with details of ER status

^*8^Proportion in total cases with details of HER2 status

^*9^Both of neoadjuvant therapy and adjuvant therapy were included.

^*10^Number and proportion in total 459 cases with details of ER status (both primary and recurrent tumor)

^*11^Among the patients with no change in their HER2 status

^*12^Details of either biopsy or excision were not available.

**Supplementary Table S5.**

Summary of the number of articles in each analysis

|  | | **The number of articles which data were available (n)** | **The number of excluded articles according to our methods**  **(n)** | **The total number of remaining articles  (n)** |
| --- | --- | --- | --- | --- |
| **OS** | **ER-loss (+/−) vs ER-concordant (+/+)** | 7 | 1 | 6 |
|  | **ER-gain (−/+) vs ER-concordant (−/−)** | 6 | 2 | 4 |
|  | **PR-loss (+/−) vs PR-concordant (+/+)** | 8 | 0 | 8 |
|  | **PR-gain (−/+) vs PR-concordant (−/−)** | 7 | 3 | 4 |
|  | **HER2-loss (+/−) vs HER2-concordant (+/+)** | 2 | 2 | 0 |
|  | **HER2-gain (−/+) vs HER2-concordant (−/−)** | 2 | 1 | 1 |
| **PRS** | **ER-loss (+/−) vs ER-concordant (+/+)** | 5 | 0 | 5 |
|  | **ER-gain (−/+) vs ER-concordant (−/−)** | 5 | 1 | 4 |
|  | **PR-loss (+/−) vs PR-concordant (+/+)** | 5 | 0 | 5 |
|  | **PR-gain (−/+) vs PR-concordant (−/−)** | 5 | 1 | 4 |
|  | **HER2-loss (+/−) vs HER2-concordant (+/+)** | 3 | 2 | 1 |
|  | **HER2-gain (−/+) vs HER2-concordant (−/−)** | 2 | 2 | 0 |

Abbreviations: ER, estrogen receptor; HER2, human epidermal growth factor receptor 2; OS, overall survival; PRS, post-recurrence survival; PR, progesterone receptor.

**Supplementary Table S6.**

Summary of survival risks of receptor discordance in the fixed-effects model, total cases, median cases, and median follow-up term

|  | Risk group | Reference group | Number of total studies (n) | HR  [95%CI] | *p*-value | I^2^ value  (%) | Statistical heterogeneity *p*-value | Risk group | | | Reference group | | |
| --- | --- | --- | --- | --- | --- | --- | --- | --- | --- | --- | --- | --- | --- |
|  |  |  |  |  |  |  |  | Total cases (n) | Median cases  (n [range]) | Median term (month [range]) | Total cases (n) | Median cases  (n [range]) | Median term  (month [range]) |
| OS | ER-loss (+/−) | ER-concordant (+/+) | 6 | **1.67**  **[1.37, 2.04]** | **<0.00001** | 23 | 0.26 | 296 | 22  [14-113] | 153  [120-209] | 870 | 149.5  [29-240] | 153  [120-315] |
|  | ER-gain (−/+) | ER-concordant (−/−) | 4 | 0.90  [0.68, 1.19] | 0.45 | **65** | **0.03** | 119 | 24.5  [11-59] | 132  [88-180] | 417 | 75  [45 - 222] | 132  [88-180] |
|  | ER-gain' (−/+) | ER-concordant’ (−/−) | 3 | 0.74  [0.54, 1.01] | 0.06 | 0 | 0.91 | 108 | 36  [13-59] | 144 [120-180] | 372 | 94  [56-222] | 144  [120-180] |
|  | PR-loss (+/−) | PR-concordant (+/+) | 8 | **1.45**  **[1.21, 1.75]** | **< 0.0001** | 18 | 0.29 | 479 | 32  [15-158] | 162  [120-198] | 614 | 69  [18-157] | 169  [120-315] |
|  | PR-gain (−/+) | PR-concordant (−/−) | 4 | 0.77  [0.59, 1.01] | 0.06 | 52 | 0.1 | 125 | 29.5  [11-55] | 150  [96-224] | 501 | 106.5  [31-257] | 150  [96-224] |
| PRS | ER-loss (+/−) | ER-concordant (+/+) | 5 | **1.72**  **[1.40, 2.11]** | **< 0.00001** | 16 | 0.31 | 278 | 29  [14-113] | 84  [60-136] | 762 | 164  [58-240] | 84  [60-177] |
|  | ER-gain (−/+) | ER-concordant (−/−) | 4 | **0.76**  **[0.59, 0.97]** | **0.03** | 0 | 0.96 | 116 | 23.5  [10-59] | 94  [60-114] | 407 | 77  [31-222] | 94  [60-114] |
|  | PR-loss (+/−) | PR-concordant (+/+) | 5 | **1.54**  **[1.27, 1.87]** | **< 0.00001** | 12 | 0.34 | 407 | 52  [25-158] | 84  [60-186] | 418 | 98  [25-157] | 84  [60-186] |
|  | PR-gain (−/+) | PR-concordant (−/−) | 4 | 1.10  [0.85, 1.43] | 0.47 | 10 | 0.34 | 116 | 25  [11-55] | 101  [60-128] | 554 | 114.5  [68-257] | 101  [60-128] |

Abbreviations: CI, confidential interval; ER, estrogen receptor; HR, hazard ratio; OS, overall survival; PR, progesterone receptor; PRS, post-recurrence survival.

Bold letters indicate statistical significance.

**Supplementary Table S7.**

Meta-regression analysis of the relationship between hazard ratios of PR-gain (−/+) and the rates of the other variables in the primary tumors

|  | Regression coefficient | SE | 95% CI | *p* value |
| --- | --- | --- | --- | --- |
| ER^*1^ | < -0.001 | 0.016 | (-0.032 to 0.031) | 0.958 |
| HER2^*2^ | 0.036 | 0.017 | **(0.003 to 0.069)** | **0.034** |
| Histologic grade^*3^ | -0.035 | 0.025 | (-0.084 to 0.015) | 0.171 |
| T factor^*4^ | 0.081 | 0.055 | (-0.026 to 0.189) | 0.138 |
| N factor^*5^ | -0.019 | 0.013 | (-0.044 to 0.006) | 0.138 |

Abbreviations: CI, confidential interval; ER, estrogen receptor; HG, histologic grade; HER2, human epidermal growth factor receptor 2; LN, lymph node; SE, standard error.

Bold letters indicate statistical significance.

^*1^The rate of ER positivity in the primary tumors for each article

^*2^The rate of HER2 positivity in the primary tumors for each article

^*3^The rate of histologic grade 3 in the primary tumors for each article

^*4^The rate of T3 or T4 in the primary tumors for each article

^*5^The rate of N1, N2 or N3 in the primary tumors for each article

**Supplementary Figure S1.**

Details of articles excluded by the 2^nd^ reviewer during screening process

**Supplementary Figure S2.**

Risk of bias using RoBANS tool

1. Risk of bias graph
2. Risk of bias summary
3. κ value for each category

(A)

(B)

(C)

| **Risk of bias assessment** | **κ value** |
| --- | --- |
| Selection of participants | 0.62 |
| Confounding variables | 0.82 |
| Measurement of exposure | 1.00 |
| Blinding of outcome assessments | 1.00 |
| Incomplete outcome data | 1.00 |
| Selective outcome reporting | 0.62 |

**Supplementary Figure S3.**

Forest plots for the comparisons of OS between receptor-loss/gain group and receptor-concordant group in the subgroup analysis according to the threshold of hormone receptor-positivity

1. Comparison of OS between ER-loss (+/−) group and ER-concordant (+/+) group
2. Comparison of OS between ER-gain (−/+) group and ER-concordant (−/−) group
3. Comparison of OS between PR-loss (+/−) group and PR-concordant (+/+) group
4. Comparison of OS between PR-gain (−/+) group and PR-concordant (−/−) group

(A)

************

(D)

(C)

(B)

**Supplementary Figure S4.**

Forest plots for the comparisons of OS between receptor-loss/gain group and receptor-concordant group in the subgroup analysis according to the assessment of risk of bias

1. Comparison of OS between ER-loss (+/−) group and ER-concordant (+/+) group
2. Comparison of OS between ER-gain (−/+) group and ER-concordant (−/−) group
3. Comparison of OS between PR-loss (+/−) group and PR-concordant (+/+) group
4. Comparison of OS between PR-gain (−/+) group and PR-concordant (−/−) group

(A)

********

(D)

(C)

(B)

**Supplementary Figure S5.**

Forest plots for the comparison of OS between the receptor-loss/gain group and receptor-concordant group in the subgroup analysis according to the study area:

1. Comparison of OS between ER-loss (+/−) group and ER-concordant (+/+) group
2. Comparison of OS between ER-gain (−/+) group and ER-concordant (−/−) group
3. Comparison of OS between PR-loss (+/−) group and PR-concordant (+/+) group
4. Comparison of OS between PR-gain (−/+) group and PR-concordant (−/−) group

(C)

(D)

(B)

(A)

**Supplementary Figure S6.**

Meta-regression analysis of the relationship between hazard ratios of PR-gain (-/+) and the rates of HER2-positive in the primary tumors

**Supplementary Figure S7.**

Funnel plots for each meta-analysis

Overall survival (OS) analysis: (A)–(D)

(A) Comparison of OS between ER-loss (+/−) group and ER-concordant (+/+) group

(B) Comparison of OS between ER-gain (−/+) group and ER-concordant (−/−) group

(C) Comparison of OS between PR-loss (+/−) group and PR-concordant (+/+) group

(D) Comparison of OS between PR-gain (−/+) group and PR-concordant (−/−) group

Post-recurrence survival (PRS) analysis: (E)–(H)

(E) Comparison of PRS between ER-loss (+/−) group and ER-concordant (+/+) group

(F) Comparison of PRS between ER-gain (−/+) group and ER-concordant (−/−) group

(G) Comparison of PRS between PR-loss (+/−) group and PR-concordant (+/+) group

(H) Comparison of PRS between PR-gain (−/+) group and PR-concordant (−/−) group

(E)

(A)

(F)

(B)

(C)

(G)

(H)

(D)

**Supplementary Text**

**Material and methods**

***Meta-analysis registration***

Our protocol was prospectively registered on PROSPERO, which is a prospective international register of systematic reviews (registration number: CRD42020161049, available from <https://www.crd.york.ac.uk/prospero/display_record.php?RecordID=161049>). Amendments of the published first protocol are available on the same site.

After registering the protocol on PROSPERO, we searched the following three electronic databases: MEDLINE, Cochrane Library, and EMBASE. Each search strategy is shown in Supplementary Table S3. We consulted with an experienced searcher (AA) to confirm the qualification of this search strategy. After merging those three search results, duplicated articles were excluded using JabRef software (<https://www.jabref.org/>) and Microsoft Excel software ver.16.40.

***Quality assessment***

All selected studies were assessed for quality independently by two authors (SS, SK) using the Risk of Bias Assessment tool for Non-randomized Studies (RoBANS)^1^, which includes six domains. Reports in which discrepancies arose between reviewers were discussed until consensus was reached. Discrepancies of quality assessment between the two independent reviewers were evaluated using the κ coefficient.

***Statistical analysis***

We used EZR software (Saitama Medical Center, Jichi Medical University, Saitama, Japan), which is a graphical user interface for R (The R Foundation for Statistical Computing, Vienna, Austria) to evaluate publication bias with Egger’s test and to perform meta-regression analysis. More precisely, EZR is a modified version of R commander designed to add the statistical functions that are frequently used in biostatistics^2^. We calculated κ value using SPSS version 27 (IBM SPSS, Armonk, NY, USA). Different ranges of κ-value were defined as follows: >0.75, excellent agreement; 0.40–0.75, fair to good agreement; and <0.40, poor agreement^3^.

References

1. Kim SY, Park JE, Lee YJ et al (2013) Testing a tool for assessing the risk of bias for nonrandomized studies showed moderate reliability and promising validity. J Clin Epidemiol 66:408–414
2. Kanda Y (2013) Investigation of the freely available easy-to-use software “EZR” for medical statistics. Bone Marrow Transplant 48:452–458
3. Fleiss JL, Levin B, Paik MC (2003) Statistical methods for rates and proportions (3^rd^ edition). John Wiley Series in Probability and Statistics
